# Airway Management Using Transnasal Humidified Rapid-Insufflation Ventilatory Exchange for Critical Distal Tracheal Stenosis Caused by a Massive Goiter: A Case Report

**DOI:** 10.7759/cureus.112899

**Published:** 2026-07-18

**Authors:** Eri Sakurai, Takahiro Nakashima, Tomoaki Saito, Tomokazu Shizuki

**Affiliations:** 1 Department of Anesthesiology, Graduate School of Medical and Dental Sciences, Kagoshima University, Kagoshima, JPN; 2 Department of Clinical Engineering, Kagoshima University Hospital, Kagoshima, JPN; 3 Department of Anesthesiology, Kagoshima University Hospital, Kagoshima, JPN

**Keywords:** airway management, apneic oxygenation, goiter, tracheal stenosis, transnasal humidified rapid-insufflation ventilatory exchange

## Abstract

Critical distal tracheal stenosis caused by a massive goiter poses a major airway challenge in which tracheal intubation and positive-pressure ventilation may fail, potentially leading to severe hypoxia during induction. We report the case of an 88-year-old woman with dementia who underwent thyroidectomy for severe distal tracheal compression caused by a large goiter. Preoperative imaging demonstrated near-critical tracheal narrowing measuring 4 × 9 mm over a 5-cm segment. Awake fiberoptic intubation was not feasible due to severe cognitive impairment and recurrent episodes of acute respiratory distress, making an awake technique dependent on spontaneous ventilation unreliable. A structured multidisciplinary airway strategy was developed, including anesthesiologists, surgeons, and clinical engineers, with a cardiovascular surgeon present in the operating room and extracorporeal life support (ECLS) prepared with immediate availability and readiness for rapid deployment as a rescue option. Within this strategy, transnasal humidified rapid-insufflation ventilatory exchange (THRIVE) was used as an oxygenation technique during apnea. Despite ineffective mask ventilation with high airway pressures, THRIVE at 70 L/min maintained oxygen saturation at 100% throughout a 12-minute apneic period and facilitated successful bronchoscopic placement of a 4.5 mm endotracheal tube beyond the stenosis. This case demonstrates that THRIVE, when used as part of a structured airway management strategy, may help maintain oxygenation in a high-risk, difficult airway where conventional ventilation techniques fail.

## Introduction

Airway management in patients with severe tracheal compression remains one of the most challenging situations in anesthetic practice. When both tracheal intubation and effective mask ventilation are difficult or fail under conventional strategies, there is a risk of severe hypoxia during induction. Although tracheal compression caused by a large goiter is relatively uncommon, severe compression can result in critical airway compromise and present a major challenge for anesthetic management. Severe distal tracheal compression represents a particularly challenging subset because the obstruction occurs beyond the larynx, where conventional airway maneuvers and standard difficult airway algorithms may be less effective [1]. In particular, marked narrowing of the distal trachea may impair both ventilation and passage of an endotracheal tube, even when airway access is successfully achieved. Transnasal humidified rapid-insufflation ventilatory exchange (THRIVE) has emerged as a valuable technique for maintaining oxygenation during apnea [2]. By delivering warmed and humidified oxygen at high flow via a nasal cannula, THRIVE prolongs safe apnea time by facilitating oxygen transfer in the absence of active ventilation. This physiological process is known as apneic oxygenation. We report a case of severe distal tracheal compression caused by a massive goiter in an elderly patient in whom conventional airway strategies, including maintenance of spontaneous ventilation and positive-pressure mask ventilation, were considered unreliable. THRIVE was used to maintain oxygenation and facilitate the safe induction of anesthesia and tracheal intubation. This case highlights the role of THRIVE as part of a structured multidisciplinary airway strategy in patients with critically narrowed distal airways.

This case was previously presented in part at the 63rd Annual Meeting of the Kyushu Society of Anesthesiology, held in Kumamoto, Japan, in 2025.

## Case presentation

An 88-year-old woman (152 cm, 56 kg) with dementia, mild aortic regurgitation, mitral valve prolapse, and congestive heart failure was scheduled for right thyroid lobectomy. She had a history of a prior left thyroid lobectomy and progressive goiter enlargement over several years.

**Figure 1:**
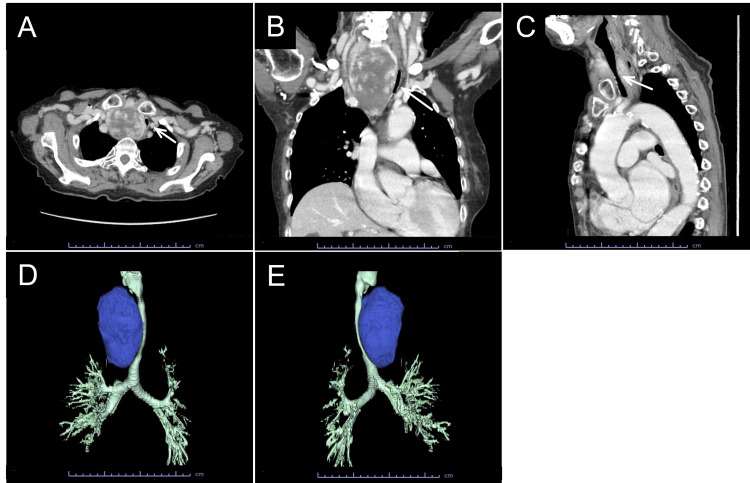
CT and three-dimensional reconstruction of a massive goiter causing severe tracheal stenosis Axial (A), coronal (B), and sagittal (C) CT images showing severe distal tracheal stenosis caused by a massive right-sided goiter. Volume-rendered (VR) anterior (D) and posterior (E) views provide three-dimensional visualization of the mass and its relationship to the trachea. White arrows indicate the critically narrowed tracheal lumen. On CT reconstruction, the stenotic segment extended from approximately 2.5 cm below the glottis to 2.5 cm above the carina, with a total length of approximately 5 cm and a minimum residual tracheal lumen of 4 × 9 mm.

In the months before surgery, she developed worsening dyspnea with repeated hospital admissions. Although the exact triggers of these episodes of acute respiratory distress could not be definitively identified, the first episode occurred during nighttime, and positional factors were considered a possible contributor. Subsequently, she developed recurrent episodes of respiratory distress triggered by mild exertion, including changing clothes and bathing. These episodes were accompanied by exertional stridor, although no stridor was present at rest. Routine airway examination revealed no apparent predictors of difficult airway management. The Mallampati classification was class II, with no limitation of mouth opening or cervical mobility. Pulmonary function testing demonstrated mixed ventilatory impairment, with a percentage vital capacity (%VC) of 63.8 %, forced expiratory volume at one second (FEV1)/forced vital capacity (FVC) of 64.7 %, and FEV1 of 0.66 L; however, interpretation was limited by her severe cognitive impairment and insufficient cooperation. Computed tomography (CT) showed a 96 × 57 × 47 mm thyroid mass causing severe tracheal compression over approximately 5 cm, with a residual lumen measuring 4 × 9 mm (Figure 1). 

Awake fiberoptic intubation was considered but deemed unsuitable because severe cognitive impairment precluded patient cooperation, and recurrent, unpredictable episodes of acute respiratory distress raised concerns regarding the reliability of a spontaneous ventilation strategy. A multidisciplinary airway plan was therefore developed involving anesthesiologists, endocrine and cardiovascular surgeons, and clinical engineers. The plan included induction with short-acting agents to permit rapid emergence if necessary, continuous THRIVE for apneic oxygenation, and immediate availability of extracorporeal life support (ECLS) as a rescue strategy.

Upon the patient's arrival in the operating room, a multidisciplinary airway and rescue plan was in place. Cardiovascular surgeons capable of initiating ECLS were present in the operating room throughout the procedure, and the ECLS circuit was prepared and immediately available. Bilateral femoral vessels were pre-assessed and prepared for rapid cannulation, allowing ECLS to be established within minutes if required. The patient's peripheral oxygen saturation (SpO₂) was 91-97% in the supine position. Preoxygenation with THRIVE at 40 L/min increased SpO₂ to 100% within two minutes. After 10 minutes, arterial blood gas analysis showed PaO₂ of 393 mmHg and PaCO₂ of 35 mmHg. Anesthesia was induced with remimazolam administered at 1.5 mg/kg/h and remifentanil at 0.3 µg/kg/min, resulting in loss of consciousness. The remimazolam infusion was then reduced to 1.0 mg/kg/h for maintenance. Rocuronium (0.6 mg/kg) was subsequently administered. THRIVE flow was increased to 70 L/min. Mask ventilation was attempted with the adjustable pressure-limiting (APL) valve set at 50 cmH₂O but remained ineffective, with only minimal chest wall movement observed on visual inspection. Flexible bronchoscopy revealed a severely narrowed but patent distal tracheal lumen. A 4.5 mm spiral endotracheal tube was successfully advanced beyond the stenosis under flexible bronchoscopic guidance. During the 12-minute period between administration of rocuronium and confirmation of tracheal intubation, SpO₂ remained at 100% under continuous THRIVE.

The surgery proceeded uneventfully. No significant airway edema was observed at the end of the procedure, and the patient was extubated safely. Postoperative recovery was uncomplicated, and she was discharged on postoperative day six.

## Discussion

This case highlights several important considerations in the management of severe distal tracheal stenosis. 

In this report, severe distal tracheal stenosis was defined as long-segment tracheal narrowing of approximately 5 cm on CT, associated with a markedly reduced residual lumen measuring approximately 4 × 9 mm. The stenotic segment involved the lower trachea and extended toward the carina, without significant involvement of the subglottic region.

First, positive-pressure mask ventilation may be ineffective in severe distal airway narrowing even at high airway pressures. Only face-mask positive-pressure ventilation was attempted, and no adjunct airway maneuvers, such as an oropharyngeal airway or a laryngeal mask airway, were used, as a bronchoscopic intubation strategy had been planned based on the anticipated distal airway obstruction. Despite attempting bag-mask ventilation with the APL valve set at 50 cmH₂O, only minimal chest wall movement was observed. This finding highlights the limited effectiveness and potential risk of conventional ventilation in this setting.

Second, although awake fiberoptic intubation is generally recommended for anticipated difficult airways [3], it may not be feasible in patients with severe cognitive impairment and unstable respiratory status. In such cases, alternative strategies and careful preoperative planning are essential. In the present case, neuromuscular blockade was administered despite anticipated ventilation difficulty because a strategy relying on maintenance of spontaneous ventilation was considered unreliable. This decision was based on recurrent, unpredictable episodes of acute respiratory distress, severe cognitive impairment precluding cooperation with awake airway management, and concerns regarding respiratory depression associated with sedative administration in the setting of critical distal tracheal stenosis. Furthermore, a predefined rescue strategy, including immediate availability of ECLS, had been established before induction, providing an additional safety margin should airway management fail.

Third, THRIVE was associated with maintenance of oxygenation throughout the apneic period despite severe distal tracheal narrowing. Although the stenosis was near-critical, flexible bronchoscopy confirmed a residual patent lumen, which may have been sufficient to permit oxygen transport during apneic oxygenation. This effect is consistent with the principle of apneic oxygenation, in which continued alveolar oxygen uptake generates a diffusion gradient that allows oxygen transfer in the absence of active ventilation [2,4-6]. The low-level positive airway pressure generated by THRIVE may also have contributed to airway patency and facilitated fiberoptic bronchoscopic navigation through the stenotic segment [4,7].

Although THRIVE has been used in a variety of difficult airway scenarios [8,9], its effectiveness in the setting of severe distal tracheal stenosis remains incompletely defined. The novelty of this case lies not in the duration of apneic oxygenation itself, but in the successful use of THRIVE in a patient with near-critical distal tracheal stenosis in whom both awake intubation and effective mask ventilation were considered unreliable. This case, therefore, suggests that THRIVE may be a useful adjunct as part of a structured airway strategy, even in advanced distal airway obstruction, when appropriate preparation and contingency planning are in place.

Importantly, oxygenation alone does not eliminate risk, as carbon dioxide (CO₂) accumulation remains a limitation of apneic oxygenation techniques. Although end-tidal CO₂ levels were acceptable in this case, more continuous monitoring, such as transcutaneous CO₂, may be useful in similar high-risk situations.

Finally, successful airway management in this case was attributable not to a single technique, but to a structured multidisciplinary strategy incorporating predefined rescue options. The use of short-acting anesthetic agents to allow rapid emergence, together with the immediate availability of ECLS, provided important safety margins should the primary plan have failed [10].

## Conclusions

Severe distal tracheal stenosis caused by a massive goiter represents a high-risk airway in which both intubation and ventilation may be difficult or fail. In this case, THRIVE was associated with maintenance of oxygenation during a prolonged apneic period and facilitated successful flexible bronchoscopic intubation despite ineffective mask ventilation. However, THRIVE should not be considered a definitive solution for airway obstruction. Its value lies in its role as part of a structured multidisciplinary airway management strategy with predefined rescue pathways. Careful preoperative assessment, appropriate patient selection, and team coordination are essential for the safe management of complex airway cases. As this report describes a single case, the generalizability of these findings remains limited, and further experience is required to determine the role of THRIVE in similar high-risk airway situations.
